# Early Workplace Communication and Problem Solving to Prevent Back Disability: Results of a Randomized Controlled Trial Among High-Risk Workers and Their Supervisors

**DOI:** 10.1007/s10926-015-9596-z

**Published:** 2015-07-23

**Authors:** Steven J. Linton, Katja Boersma, Michal Traczyk, William Shaw, Michael Nicholas

**Affiliations:** Department of Law, Psychology, and Social Work, Center for Health and Medical Psychology, Örebro University, Örebro, Sweden; Landstingshälsan, Occupational Health Services, Örebro, Sweden; Center for Disability Research, Liberty Mutual Research Institute for Safety, Boston, MA USA; Pain Management Research Institute, University of Sydney at Royal North Shore Hospital, Sydney, Australia

**Keywords:** Prevention, Screening, Randomized controlled trial, Back pain, Problem solving, Communication skills, Early intervention, Work absence

## Abstract

*Purpose* There is a clear need for interventions that successfully prevent the development of disability due to back pain. We hypothesized that an intervention aimed at both the worker and the workplace could be effective. Hence, we tested the effects of a new early intervention, based on the misdirected problem solving model, aimed at both workers at risk of long-term impairments and their workplace. *Methods* Supervisors of volunteers with back pain, no red flags, and a high score on a screen (Örebro Musculoskeletal Screening Questionnaire) were randomized to either an evidence based treatment as usual (TAU) or to a worker and workplace package (WWP). The WWP intervention included communication and problem solving skills for the patient and their immediate supervisor. The key outcome variables of work absence due to pain, health-care utilization, perceived health, and pain intensity were collected before, after and at a 6 month follow up. *Results* The WWP showed significantly larger improvements relative to the TAU for work absence due to pain, perceived health, and health-care utilization. Both groups improved on pain ratings but there was no significant difference between the groups. The WWP not only had significantly fewer participants utilizing health care and work absence due to pain, but the number of health care visits and days absent were also significantly lower than the TAU. *Conclusions* The WWP with problem solving and communication skills resulted in fewer days off work, fewer health care visits and better perceived health. This supports the misdirected problem solving model and indicates that screening combined with an active intervention to enhance skills is quite successful and likely cost-effective. Future research should replicate and extend these findings with health-economic analyses.

## Introduction

Musculoskeletal disorders such as back or neck pain continue to be frequent and costly problems and a leading cause of functional difficulties including work impairment [1–7]. While the literature base establishing risk factors for long-term problems has grown, this knowledge is under-utilized in practice, and there remains a need for effective early intervention strategies tailored to address these risk factors [8, 9]. One implementation challenge has been the need to capture the synergy of both individual-level and organizational strategies to prevent long-term back disability, and there have been few efforts to integrate interventions with workers and employers in a single trial. Instead, most programs for early intervention are offered in a primary care facility and focus exclusively on the individual [9]. Currently, risk screening and early intervention is left to a single health-care provider with limited time and training to deal with workplace and psychosocial concerns [8]. This study is a randomized investigation of a new approach to back disability prevention that applies psychological theories of pain management and focuses these efforts on both the worker and the workplace.

A number of self-report scales have been shown to predict those at greatest risk of developing long-term musculoskeletal pain-related impairments in primary care [10, 11]. For example, the Örebro Musculoskeletal Pain Screening Questionnaire (ÖMPSQ) predicts lengthy claims and high costs, even when used at the first visit or as a survey of workers reporting pain [10, 12–16]. Second, there is evidence that various cognitive-behavioral preventive interventions reduce the risk of long-term work impairment [9, 17–22]. Third, brief workplace interventions focusing on supervisors have also shown benefits for reducing disability outcomes. Even though supervisors play a key role, they commonly report a lack of training in dealing with employees with pain problems [23–25]. Accordingly, programs that teach supervisors basic skills (e.g., communication and negotiating accommodations) may have significant benefits for workers with pain problems [24, 26–28].

Based on the existing evidence supporting patient screening, early intervention, and supervisor involvement, we developed an experimental intervention that was based on two current theoretical perspectives. First, we focused on problem-solving [29], a fundamental component of evidence-based cognitive-behavioral treatment strategies in psychology [29–33]. Since there are a large variety of risk factors and potential problems for individuals with back pain, solving relevant lifestyle problems is vital, and this method allows for adaptation to the needs of the individual patient. Second, we employed elements of the misdirected problem solving model. The fundamental idea of this model is that patients expend all of their problem solving efforts searching for a cure for pain, and repeated failures can inadvertently increase worry and distress [34]. Efforts to redirect problem solving efforts toward lifestyle challenges may improve coping.

While most psychosocial intervention strategies have been focused on pain sufferers, it’s possible that interventions directed to other supporting individuals (e.g., spouses, family members, co-workers, supervisors) may also help to overcome problems associated with pain. Therefore, we developed a program that includes an intervention at the workplace by providing a brief training program for supervisors. In this study, we evaluate whether it is effective to focus on communication and problem-solving with workers with back pain and their supervisors.

## Aim

The purpose of this study was to test the effects of an early worker and workplace intervention program for employees at risk of developing long-term work impairments due to back pain. To this end, we compared this program with treatment as usual (based on best practice recommendations) in order to study its effects on outcomes e.g. work absence due to pain, perceived health, health care utilization, and pain.

## Methods

### Overview of the Design

We conducted a 2-arm randomized parallel controlled trial comparing: (1) a worker and workplace treatment package (WWP) or, (2) treatment as usual (TAU) based on current guidelines. Our main outcome variables were absence due to pain, health care utilization, perceived health, and pain intensity ratings. The initial screening and pre-treatment (baseline) assessment was followed by a treatment phase (4 weeks), and then a post-treatment and 6 months follow-up assessment. The design and procedures followed the guidelines formulated by the CONSORT group [35]. The Regional Ethics Board approved the study.

### Participants

#### Recruitment

Participants were recruited through the occupational health care service via invitations provided at workplaces as well as via screening when workers sought care at a single occupational health care center. To be included in the study, participants needed to fulfill the following criteria: (1) suffering from musculoskeletal low back pain, (2) elevated risk (>40) for developing chronic pain problems according to the the Örebro Musculoskeletal Pain Screening Questionnaire, short form (ÖMPSQ-SF) [12], (3) no red flags (signs of a possibly serious underlying condition), and (4) consenting to have their supervisor contacted for participation in the study.

**Fig. 1:**
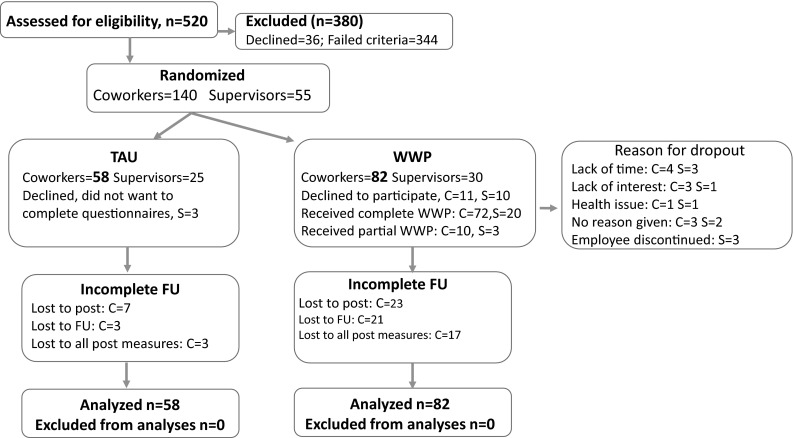
A flow chart of recruitment and participants over the course of the study. "C" denotes coworkers and "S" supervisors

Figure 1 provides a flow chart of the recruitment procedure. A total of 520 employees reported their interest in participation in the trial and were assessed for eligibility. The assessment procedure was two-step. First, employees filled out a screening questionnaire to assess the risk of developing chronic pain. If the employee reported back pain and had a screening score above 40 (N = 163), they were invited to continue the assessment with a clinical interview. The clinical interview included an evaluation of possible “red flags” and this resulted in two individuals being excluded from the study and referred for further medical examination. The remaining employees were invited to participate in the study and asked for explicit consent to contact their workplace supervisors. When contacted to schedule participation, an addition 36 individuals were no longer suffering pain and declined the offer. This left 140 participants for the present trial. However, when supervisors were contacted, 7 declined the invitation mostly due to time limitations (the employee was provided with the treatment, but excluded from the study). Of the 30 supervisors participating in the WWP intervention, 20 completed the entire training, while 10 completed some training (see Fig. 1).

#### Sample

Partakers in the study were 55 immediate supervisors and their 140 volunteer employees (132 (94 %) women) aged between 27 and 65 years, working predominantly within health care, social services and education (e.g. nurses, health care assistants, teachers, administration staff, home care personal). An overview of the characteristics of the employees is provided in Table 1. The length of employment at the current workplace varied between 1 and 32 years. The majority (72.9 %) of the participants reported pain symptoms of more than 1 year, while 12.9 % reported pain symptoms of less than 6 months. Multiple pain sites (two or more) were much more common (80.7 %) than pain localized to a single body part (19.3 %). Participants scored their current pain intensity (last week, range 0–10) at a mean of 5.9 (SD = 1.9) and their average pain intensity (last 3 months, range 0–10) at 6.27 (SD = 1.6). All participants, as required by the inclusion criteria, reported an elevated risk of developing long-term pain disability with the mean ÖMPSQ_short_ score being 54.94 (SD = 9.6) where a score of 40 signifies ‘moderate risk’ and a score of 50 signifies ‘high risk’ [12]. Half of the study sample reported at least one episode of work absence due to pain during the 3 months prior to the treatment due to the pain. Within this time frame 42.9 % of participants had sought help for their pain problems on at least one occasion. Finally, participants rated their mean general health status at 62 points (SD = 16.6) on a 100-point scale. There were no significant differences in any of these variables at the pre test.

**Table 1:** Pre test characteristics of the two groups of employees

Variable	WWP	TAU	Test statistics (*F* or χ^2^)
N	82	58	
Age (years, *M*)	49.65 (9.98)	49.90 (10.38)	*F*_(1,138)_ = 0.876, *ns*
Gender *n* (% Woman)	78 (95.1 %)	54 (93.1 %)	χ_(1)_^2^ = 0.257, *ns*
Origin *n* (% Swedish)	72 (87.8 %)	54 (93.1 %)	χ_(1)_^2^ = 1.060, *ns*
Employment (years, *M*)	14.57 (10.55)	13.00 (10.28)	*F*_(1,136)_ = 0.755, *ns*
Number of pain sites *n* (%)			χ_(4)_^2^ = 1.139, *ns*
1	14 (17.1 %)	13 (22.4 %)	
2	27 (32.9 %)	19 (32.8 %)	
3	26 (31.7 %)	16 (27.6 %)	
4	8 (9.8 %)	4 (6.9 %)	
5	7 (8.5 %)	6 (10.3 %)	
Pain duration			χ_(2)_^2^ = 0.717, *ns*
<6 months	11 (13.4 %)	7 (12.1 %)	
6 months–1 year	10 (12.2 %)	10 (17.2 %)	
>1 year	61 (74.4 %)	41 (70.7 %)	
ÖMPSQ_short_ (*M*)	55.45 (9.28)	54.24 (10.08)	*F*_*(*1,138)_ = 0.552, *ns*

Values in parentheses are standard deviations

*WWP* worker and workplace package, *TAU* treatment as usual. *ÖMPSQ* Örebro Musculoskeletal Pain Screening Questionnaire, *ns* non-significant (*p* > .05)

#### Randomization

In order to prevent the contamination of the interventions that would occur if a supervisor would have employees participating in both groups, we randomized supervisors to either TAU or WWP using a computer generated randomization procedure with a one to one allocation. Thus, when an eligible employee volunteered for the study and their supervisor agreed to participate, *the supervisor* was randomly allocated to a group by opening the consecutively numbered, sealed opaque envelope. If the employee’s supervisor was already in the study, the employee was therefore also assigned to the supervisor’s group allocation. This ensured that a supervisor could only be involved in one arm of the study and resulted in n = 58 participants assigned to TAU and n = 82 assigned to the WWP.

### Interventions

After allocation, the participants began the treatment within 2 weeks. All interventions and data collection took place at a medium-sized Swedish occupation health care center (Landstingshälsan, Örebro) mainly serving municipal and county council employees. Psychologists employed at this center delivered the treatment.

#### Treatment as Usual (TAU)

Care was provided according to the latest evidence-based guidelines and at the discretion of the health care professionals at the center. This involved one or more of the following: physical examination, consultation with a nurse, physician, psychologist, or physical therapist, guided physical activity, physical therapy, participation in self-help or educational courses. The study made no recommendations or restrictions. Additionally, participants, at their own discretion, were free to seek other health care providers. As a part of the routine care provided by the occupational health care service, each participant’s supervisor was contacted to provide feedback and possible advice.

#### Worker and Workplace Package Treatment (WWP)

Participants in this group received a manualized, short-term, preventive intervention based on cognitive behavioral principles. The overarching goal of the worker intervention was to increase their ability to self-manage daily work-related obstacles related to their pain experience. The main goal of the supervisor intervention was to minimize the impact of workplace-related psychosocial risk factors for developing chronic pain problems and to create a supportive work environment. Considering the brief character of the intervention, the supervisors were also offered the opportunity to telephone or e-mail consultation, if needed, within 2 months after the last session. Four clinical psychologists delivered the treatment after they had completed a theoretical and practical training course in the method (16 h) provided by the research team. Three of these therapists were licensed and one was undergoing a clinical internship as the last step for licensing. An overview of the structure and content of the WWP is described in Table 2. Communication training was based on empathetic, “person-centered” techniques where the principles of validation served as a basis [36, 37]. Problem-solving skills training was based on the successful programs described in the literature [38–40].

**Table 2:** An overview of the interventions provided for the worker (patient) and the workplace (supervisor)

“Worker” (patient) intervention			“Workplace” (supervisor) intervention		
Session theme	Content	Focus	Session theme	Content	Focus
I. Problem analysis and goal setting (60–90 min)	Validation Motivational interviewing Psycho education (biopsychosocial model, fear avoidance and misdirected problem solving) Valuing and goal setting Homework	To reframe the problem definition from pain as the main problem to be solved, to pain as an obstacle to obtaining long-term goals. Usually redefinition meant transitioning from problem statements such as: “My pain is my problem” to statements such as: “I want to do X, but my pain is hindering me”	I. Problem analysis and developing problem solving skills (90–120 min)	Validation Psycho education (biopsychosocial model, misdirected problem solving, influence of psycho social work factors) Problem solving skills training Homework	To identify difficulties that may arise when someone in the staff suffers from pain. To inform about the biopsychosocial model of pain and on how work factors may influence pain problems. To introduce the misdirected problem-solving model and train problem-solving techniques
II. Developing problem solving skills (60–90 min)	Problem solving skills training Home work	To train problem solving to help reach valued goals	II. Effective communication (90–120 min)	Communication skills training	To practice responding to employees’ pain behaviors in a validating way; establishing effective problem solving
III. Effective communication at work (60–90 min)	Motivational interviewing Communication skills training Role play Homework	To develop skills for assertive communication about pain-related experiences, feelings, and needs. To train to communicate with key individuals at the work place so as to increase likelihood of a joint effort to solve the current pain-related problems and arrive at mutually acceptable compromises	III. Follow-up and troubleshooting	Telephone or email contact to obtain feedback and troubleshoot any problems or discuss issues that arose	Application of learned skills

After the treatment was completed, all participants received the post-test questionnaires together with a small incentive (movie ticket) via postal mail. A reminder was sent in case participants did not return the questionnaires within 1 week. If they still did not respond, participants were reminded to fill in the questionnaires by phone by a research assistant. A similar procedure was used to collect the follow-up assessment 6 months after the treatment.

### Measures

#### Pain Intensity

Pain intensity was measured with two items from ÖMPSQ [12]: “How would you rate the pain that you have had during the past week?” and “In the past 3 months, on average, how bad was your pain?” The items use 0–10 response scales, where higher ratings indicate more intense pain. The ÖMPSQ, including the individual pain items, has shown good validity and reliability [10, 41]. The measure was used at pretest and follow-up.

#### Work-Absence

Participants provided reports of their work absence due to pain on the following items: “Have you been off-work due to pain during the past three (3) months?” and “How many days have you been on sick-leave due to pain?” This method has shown good reliability and validity as compared to official records [42, 43]. The measure was used at pretest and the follow-up.

#### Health-Care Utilization

Utilization of health-care services was assessed with the question: “Have you sought help for your pain problems?” [44, 45]. If the answer was yes, supplementary information about the number of visits to various health-care providers (nurse, physical therapist, general practitioner, specialist, other) was obtained.

#### Perceived Health

Participants’ perceived health status was measured on a visual analogue scale (VAS) from 0 (worst possible health status) to 100 (best possible health status). The scale was presented in a vertical format and patterned after the EQ-5D [46]. This scale has good psychometric properties [47].

### Statistical Analyses

Data analyses were conducted using SPSS 21.0 statistical software (IBM Corp., Armonk, NY, released 2012). For binary outcome variables (work absence due to pain and health care utilization) logistic regression analysis was used. To analyze the continuous outcome variable (perceived health) linear mixed model methods with maximal likelihood estimation was used [48] (Raudenbush & Bryk, 2002; Tabachnick & Fidell, 2012). Analyses were carried out using an intention-to-treat approach employing the last observation carried forward imputation to account for missing data. An analysis of non-responders showed no significant differences between participants who dropped out from the study and those who were retained. Since the analyses of pretest scores showed no significant differences between the groups (see Tables 1 and 3), the results focus on differences in outcome between the groups. For the variables of absence due to pain and health care utilization we have examined both the percentage of participants in each group involved as well as the number of day off work and the number of health care visits since the data is skewed. Thus, we capture how many workers were off work or used health care as well as the number of times they utilized these benefits.

**Table 3:** Means and standard deviations or percentages for outcome variables per treatment group over the course of the study and tests of significance

Variable	Worker and workplace package			Treatment as usual			Statistical comparisons		
	Pre-test	Post-test	Follow-up	Pre-test	Post-test	Follow-up	Pre-test	Post-test	Follow-up
Pain intensity (*M*)									
Past week	5.80 (1.98)		4.69 (2.49)	6.14 (1.90)		5.24 (2.30)	*F*_(1,138)_ = 0.997, *ns*		*F*_(1,138)_ = 1.744, *ns*
Past 3 months	6.28 (1.61)		5.30 (2.19)	6.26 (1.48)		5.48 (1.94)	*F*_(1,138)_ = 0.016, *ns*		*F*_(1,138)_ = 0.246, *ns*
Work absence, *n* (% yes)	38 (46.3 %)		17 (20.7 %)	32 (55.2 %)		23 (39.6 %)	χ_(1)_^2^ = 1.060, *ns*		χ_(1)_^2^ = 5.961*
Health care, % yes (*n*)	45 % (*n* = 34)	27 % (*n* = 22)	28 % (*n* = 23)	40 % (*n* = 23)	48 % (*n* = 28)	57 % (*n* = 33)	χ_(1)_^2^ = 0.415, *ns*	χ_(1)_^2^ = 6.806*	χ_(1)_^2^ = 11.780**
Perceived health (*M*)	60.23 (16.67)	69.52 (17.32)	72.76 (16.17)	64.40 (16.28)	60.72 (20.16)	59.02 (19.50)	*F*_(1,138)_ = 2.161, *ns*	*F*_(1,138)_ = 7.648*	*F*_(1,138)_ = 20.642**

Values in parentheses are standard deviations. Test statistics: one-way ANOVA (*F*) or chi square (χ^2^)

*ns* non-significant

* *p* < .05; ** *p* < .01

## Results

**Fig. 2:**
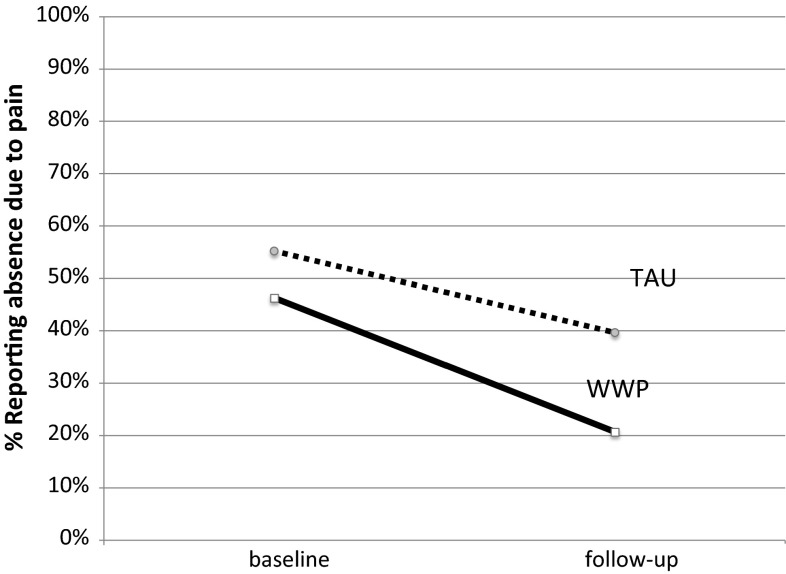
Proportion of participants reporting work absence due to pain at baseline and follow-up. *TAU* treatment as usual, *WWP* worker and workplace package

An overview of the results on the outcome variables is provided in Table 3 including means, standard deviations, percentages at the pretest, posttest and 6 month follow up as well as the statistical comparisons. Work absence due to pain is illustrated graphically in Fig. 2 which shows the percentage of group members off work due to pain before the interventions and at the follow up. The figure shows that there were fewer incidents of work absence due to pain at follow up as compared to the pretest period in both treatment arms. However, the WWP treatment arm showed greater improvement and more than halved incidence. A logistic regression was calculated where treatment group was used as the predictor variable and work absence due to pain at baseline as a control variable. The result showed a significant difference between the treatment groups. Comparison of the full model against the constant only model was significant [χ^2^ (2) = 22.565, *p* < .001] indicating that predictors included in the model explained pain related sick absence at follow-up. The model fit assessed by Homer and Lemenshow test was good [χ^2^ (2) = 0.694, *p* = .707] with Nagelkerke R^2^ = .213. The analysis showed that for participants receiving TAU the risk of reporting work absence due to pain was nearly 2.5 times higher than for participants in the WWP treatment arm [β(SE) = 0.893(0.408), OR(95 %CI) = 2.44 (1.10–5.43), *p* < .05]. In addition, there was also a significant difference in the number of days off work as the TAU had a mean of 15.4 days off work while the WWP had a mean of 4.1 days (t = 2.23, *df* = 115, *p* = .028).

**Fig. 3:**
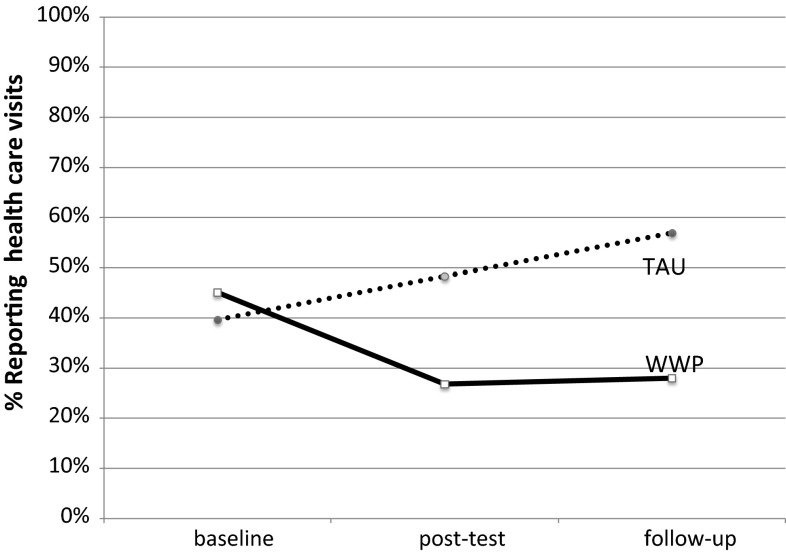
Proportion of participants reporting visits at health care providers due to pain over time. *TAU* treatment as usual, *WWP* worker and workplace package

The proportion of participants seeking health care at baseline, post-test and follow-up are graphically depicted in Fig. 3. As can be seen, the number of people who sought care for pain increased continually in the TAU arm while in the WWP arm there was a marked decrease at post-test and follow up as compared to the baseline. A logistic regression on health care utilization at follow up with treatment as a predictor, controlling for health care utilization at baseline showed that there were significant differences in outcome between the treatment arms. Comparison of the full model against the constant only model was significant, [χ^2^ (2) = 24.194, *p* < .001] indicating that predictors included in the model explained health care utilization at follow-up. The model fit assessed by Homer and Lemenshow test was acceptable [χ^2^ (2) = 0.069, *p* = .966] with Nagelkerke R^2^ = .215. The analysis showed that for participants receiving TAU the probability for seeking health care was more than 4 times higher than for participants in the WWP group [β(SE) = 1.426(0.393), OR(95 %CI) = 4.16 (1.10–8.99), *p* < .001]. There was also a difference in the number of reported health care visits at follow-up. The TAU had a mean of 3.1 visits while the WWP had a mean of 1.2 (t = 2.92, *df* = 112, *p* = .004).

**Fig. 4:**
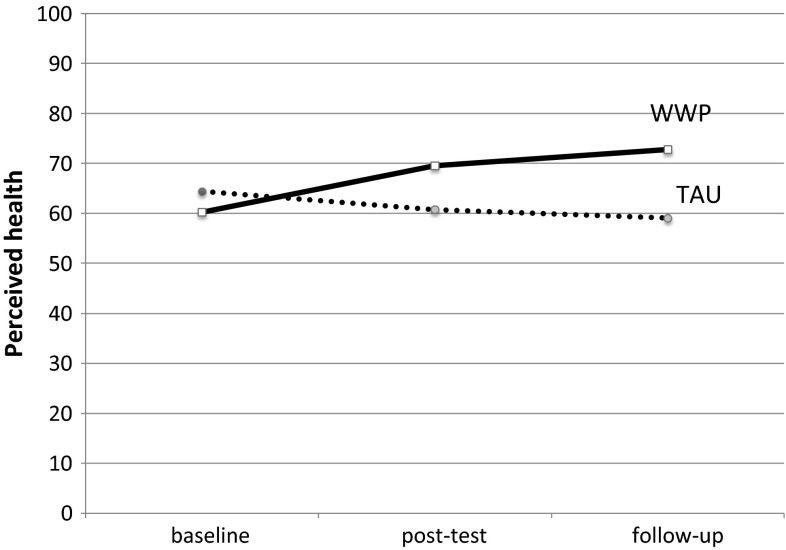
Reported health status over the time of the study. *TAU* treatment as usual, *WWP* worker and workplace package

Changes in perceived health scores over the course of the study are shown in Fig. 4. Participants in the WWP group rated improvements in their health from baseline to post-test and again, though less incremental, between post-test and follow-up. In contrast, perceived health scores in the TAU arm decreased slightly over time. In order to investigate differences in outcome on perceived health, a mixed models analysis was used with treatment, time (in months) and treatment-time interaction as predictors of perceived health at follow-up. The full model was tested against an intercept-only model showing significant improvement in data-fit [χ^2^ (4) = 182.11, *p* < .001]. The results also showed a significant time × treatment interaction effect [F(1,280) = 35.692, *p* < .001] indicating significant differences in perceived health between the treatment arms across time. Indeed, this interaction analysis showed that participants in the WWP treatment arm reported greater improvement in perceived health over time as compared to the TAU treatment arm.

Finally, pain ratings were evaluated. Both groups rated their pain at about 6 at the baseline for the past week as well as the past 3 months. Moreover, both groups rated less intense pain at the follow up with reductions of approximately 1 point. However, the difference between the groups was not significant.

## Discussion

This study found that a brief intervention focusing on problem-solving and communication skills for both the worker and the workplace resulted in significantly better improvements in perceived health, health-care utilization, and even work absenteeism due to pain as compared to a TAU based on current guidelines. Indeed, half as many of those receiving the WWP reported work absence due to pain, and these people reported less than a third as many days as compared to the TAU. Similarly, half as many in the WWP reported continued heath care visits and these participants reported half as many visits as did the TAU. This outcome provides some support that participants were able to (re)frame the problem as one of lifestyle limitations and coping challenges rather than as simply the need to find a cure. Consequently, this study provides a potentially new and feasible strategy for engaging both workers and their workplace in early pain management interventions.

Our study has several implications for theory as well as the clinic. While our findings are in line with earlier literature showing that psychologically informed early interventions are effective [9], a first novelty is that this study succeeded in combining interventions aimed at the worker and the workplace. Thus it provides impetus for actively providing this type of program for workers and their supervisors. The worker and the supervisor both participated in the same type of intervention which we hypothesized would result in improved communication and problem solving capabilities. Both the workers and the supervisors reported that they valued the program and recommended it to be continued. Participation from supervisors and workers was high. Moreover, the intervention was also acceptable to the various health care professionals at the occupational health care facility thus uniting these resources and enhancing implementation. Indeed, the problem solving involved a variety of solutions ranging over the entire biopsychosocial range. Future research will need to study what specific effects the program had on problem solving for both patients and their supervisors. Additional research is needed to specifically isolate how patients and supervisors interact before and after the intervention to determine whether the results have been directly influenced by them problem solving together.

A second novelty is the method of identifying participants. Rather than relying on clinical judgment or the passing of time, we judiciously utilized scores on the ÖMPSQ-SF. We provided the screen via a survey at work or at the first visit to the center. Thus, it was employed systematically and further intervention was guided by the score. This time frame was chosen to avoid over-medicalization of the pain problem. However, the current study shows that participants nevertheless had experienced a problem (off-and-on) for some time with more than 85 % reporting some problem for more than 6 months and about a third reporting a history of medical care and/or work absence due to pain. This may reflect a fallacy in the literature and clinic that patients seeking care for a new episode constitutes “acute” pain. More likely, it constitutes recurrent or persistent pain [49]. Similarly, a large trial that recruited patients in primary care had just 17 % with a pain duration of less than 1 month and nearly a majority (46 %) having had pain 6 months to over 3 years [22]. So, while there has been a fear that intervening too early might over-pathologize the problem and make it worse, our data suggest that this intervention is effective for those reporting a high score on the screening tool.

A third novelty with the present study is that it is the first trial to our knowledge of an intervention that is based on the misdirected problem solving model. Our results support the utility of the model, and suggest that how patients and supervisors formulate the problem may have significant impact on outcome. Our experiences during the trial suggest that the model is acceptable to health care personnel as well as to clients and supervisors. Although clients often anticipated medical interventions, they reported understanding the ideas involved and participation rates were very high. Patients often reported discovering that the pain was not the only or even the main problem and they enjoyed working toward clear-cut goals.

An interesting finding is that while the groups differed on several important outcomes (e.g., work absence due to pain), they did not differ on pain intensity ratings. Both groups improved on pain intensity ratings, but there was no significant difference between the groups. This indicates that significant reductions in work absence due to pain, improvements in overall health, and the need for less health care are not strictly dependent on pain intensity. This reinforces the idea that early interventions may well focus on reducing the *impact* of the pain rather than simply the *intensity* of the pain. Further, it also implies that the WWP intervention was successful in framing the problem in a broad (and solvable) fashion. Indeed, patients often worked on solving problems related to their function (being able to work or participate in desired activities).

The feasibility of providing early intervention is dependent on its cost-benefit. We provided a screening procedure and those at risk received 3 sessions of intervention and their supervisor 2 sessions (plus email and telephone support). Based on the time required to administer the program (coordinating, screening, psychologist time) as well as overhead charges, we estimate the cost at 10 000SEK (currently $1150) per participant in the WWP. A major benefit is the reduction of work absence due to pain where the WWP group had 11.3 fewer days than the TAU over a 6-month period. Using an average compensation level (1000:sek/day) and an estimated cost for the workplace (500:/day) in lost productivity and administration, the benefit is 16,950:SEK (currently $1949). This is an immediate savings of 6950 SEK ($800) per participant over half a year’s time and previous research has shown that such difference tend to last for at least 5 years [50]. Since this is not a proper health-economic evaluation, future research should include such evaluations.

While this study has featured several initiatives it also has limitations that need to be kept in mind when interpreting the results. The worker and workplace intervention was tested as a package and thus we do not know which components were most effective or necessary. Our design permitted comparisons with recommended treatment, but additional investigations are needed to establish the contribution of the elements of the package. We did not study possible mediators of the effects and future studies using repeated measures during treatment will be needed to test the role of validation and problem solving on outcome. Although the intervention was designed to enhance communication and problem solving between the supervisor and the worker, we were unable to assess this. Our data collection focused on the individual workers suffering pain and a limitation is the lack of data about the supervisors and how they perceived and utilized the intervention. Future work would benefit from tracking supervisors and workers specifically assessing application. However, this study does demonstrate an effect of the interventions and therefore further research is warranted with additional measures and designs.

Our results suggest that how patients and supervisors frame, communicate about, and solve problems is important for outcome. Providing a short training in communication and problem solving skills was successful in engaging both the worker and the supervisor and resulted in significant improvements in self-reported health as well as work absenteeism due to pain. Developing this intervention and implementing it in primary care may provide a much needed step forward in preventing persistent work impairment due to back pain.
